# The Medical versus Zoological Concept of Outflow Tract Valves of the Vertebrate Heart

**DOI:** 10.3390/jcdd9100318

**Published:** 2022-09-22

**Authors:** Valentín Sans-Coma, Bárbara Pozo-Vilumbrales, María Carmen Fernández, Miguel Á. López-Unzu, María Teresa Soto-Navarrete, Ana Carmen Durán, Josep M. Arqué, Borja Fernández

**Affiliations:** 1 Departamento de Biología Animal, Facultad de Ciencias, Universidad de Málaga, 29071 Málaga, Spain; 2 Instituto de Investigación Biomédica de Málaga (IBIMA), Universidad de Málaga, 29071 Málaga, Spain; 3 Departamento de Anatomía Humana, Medicina Legal e Historia de la Medicina, Facultad de Medicina, 29071 Málaga, Spain; 4 Centro Nacional de Investigaciones Cardiovasculares (CNIC), 28029 Madrid, Spain; 5 Departamento de Cirugía Cardiaca, Hospital Quirón, 29004 Málaga, Spain; 6 Centro de Investigación Biomédica en Red, Enfermedades Cardiovasculares (CIBERCV), 28029 Málaga, Spain

**Keywords:** heart, outflow tract valves, vertebrates, medical concept, zoological concept

## Abstract

The anatomical elements that in humans prevent blood backflow from the aorta and pulmonary artery to the left and right ventriclesare the aortic and pulmonary valves, respectively. Each valve regularly consists of three leaflets (cusps), each supported by its valvular sinus. From the medical viewpoint, each set of three leaflets and sinuses is regarded as a morpho-functional unit. This notion also applies to birds and non-human mammals. However, the structures that prevent the return of blood to the heart in other vertebrates are notably different. This has led to discrepancies between physicians and zoologists in defining what a cardiac outflow tract valve is. The aim here is to compare the gross anatomy of the outflow tract valvular system among several groups of vertebrates in order to understand the conceptual and nomenclature controversies in the field.

## 1. Introduction

The anatomical elements that in humans guard the unidirectional blood flow from the cardiac ventricles to the aortic and pulmonary arteries are the arterial (semilunar) valves. The valve that prevents blood backflow from the aorta to the left ventricle is the aortic valve, while that which performs this function between the pulmonary artery and the right ventricle is the pulmonary or pulmonic valve. The main medical interest in arterial valves is that their congenital malformations and diseases over a lifetime are clinically relevant [1,2,3]. Although both valves are subject to similar complications, those affecting the aortic valve cause the most severe effects [4,5,6,7].

**Figure 1:**
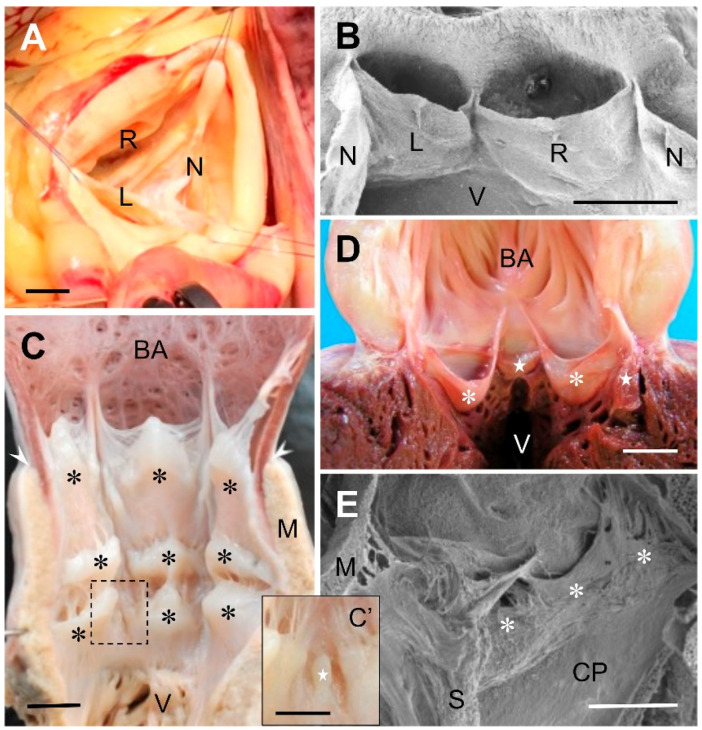
(**A**) Surgical view of a human normal, tricuspid, aortic valve. (**B**) Scanning electron micrograph of the aortic valve of a Syrian hamster (*Mesocricetus auratus*). The valve has been opened through the non-coronary sinus to expose the anterior aspect of the valve. (**C**) Ventral open-cut view of the cardiac outflow tract of the thresher shark, *Alopias vulpinus*. The conus arteriosus is furnished with three transverse rows of valves (asteriks), distal, intermediate, and proximal. The rows have three valves each, of similar size. The valves of the distal row are bigger. In the intermediate and proximal rows there are additional valves very reduced in size, ((**C’**), inset). The arrowheads indicate the anterior limit of the conus myocardium. (**D**) Ventral open-cut view of the cardiac outflow tract of the Atlantic bluefin tuna, *Thunnus thynnus*. A single row of four valves, two major (asterisks) and two minor (stars), similar in shape to human valves, are located in the conus, at the base of the bulbus arteriosus (BA). (**E**) Scanning electron micrograph of the distal region of the cardiac outflow tract of the African clawed frog, *Xenopus laevis*. The open-cut view shows a set of pocket-like valves (asterisks) located at the distal end of the *cavum pulmo-cutaneum* (CP). L, left coronary leaflet; M, myocardium; N, non-coronary (dorsal, posterior) leaflet; R, right coronary leaflet; S, spiral valve; V, ventricle. Scale bars: (**A**): 1 cm; (**B**–**E**): 0.5 mm; (**C’**): 0.25 mm.

The normal (tricuspid or trifoliate) condition of both the aortic [8,9,10,11,12] and pulmonary [9,13,14] valves is characterized by the presence of three leaflets (cusps) of similar size, each supported by its valvular sinus (Figure 1A,B). The leaflets are the most mobile components of the valve, which open and close during the cardiac cycle. The sinuses are the portions of the arterial roots to the borders of which the leaflets attach following a parabolic line. The attachments of adjacent leaflets to the sinus walls join distally at three points named commissures. The attachments of the leaflets diverge from the commissures towards the ventricle. As a result of this divergence there is a triangular space between adjacent leaflets called the subvalvular fibrous interleaflet triangle [15]. Thus, in each valve there are three interleaflet triangles. The distal vertex of each triangle is the point at which two adjacent leaflets join to form a commissure.

Regarding the whole aortic root, which is the conduit between the left ventricle and the ascending aorta that contains the aortic valve [16], there is still no definitive consensus on how to best define its anatomical components. The variety of responses given by several cardiothoracic surgeons to the questionnaire proposed by Sievers et al. [17] in an attempt to standardize the nomenclature of the aortic root already illustrates the significant discrepancies existing on this issue. Recently, a thorough study by Michelena et al. [18] has provided valuable information that favors the integration of criteria on the part of the various specialists in human hearts and, especially, on the bicuspid or bifoliate condition of the aortic valve. Possibly, not all the questions that remain up in the air have been resolved, but the progress that has been made is highly significant.

Beyond these controversies, the fact that we will emphasize here is that each of the complex structures located in the aortic root and at the base of the pulmonary artery preventing blood backflow to the ventricles is regarded by physicians as a valve, and not as a set of valves. This notion also applies to avian [19,20,21,22,23] and mammalian [24,25,26,27,28,29,30,31] species used as animal models in embryological, pathological, and genetic studies of the arterial valves (Figure 1B).

In contrast, the structures that prevent the return of blood to the heart in other groups of vertebrates, referred generally to as outflow tract valves of the heart, diverge notably from those of mammals and birds. The differences concern the number, shape, size, and spatial arrangement of the valves. This leads to disagreements between physicians and zoologists in defining what a cardiac outflow tract valve is.

The purpose here is far from suggesting any change that might affect the medical notion of arterial valves. It is well known that the earliest description and drawings of the anatomy of the human aortic valve stem from Leonardo da Vinci in the sixteenth century (see [32]). Thus, any attempt to modify concepts that have been consolidated over so many years, even though some of them are still controversial, would be inappropriate. The aim is to compare gross anatomically the cardiac outflow tract valves of several zoological groups to contribute to a better understanding and clarification of the conceptual and nomenclature discrepancies which persist between specialists, and which often cause confusion to scholars and students who are interested in the anatomy of the vertebrate heart.

## 2. The Cardiac Outflow Tract Valves of Chondrichthyans and Actinopterygians

The first relevant anatomical study of the cardiac outflow tract valves of chondrichthyans (cartilaginous fishes) and phylogenetically early actinopterygians (ray-finned fishes) was that of Stöhr [33]. In both groups, the outflow tract valves, located at the luminal side of the myocardial conus arteriosus, are usually termed conal or conus valves. Stöhr identified different valve morphologies, which he classified into four types, namely, pocket-like valves (‘Taschenklappen’), small tongue-like valves (‘Querleisten’), rudimentary valves (‘Zwischenklappen’) and knot-like valves (‘Knötchen’). It should be noted that at that time, the term valve referred exclusively to the membranous structure that closes temporarily a part of the conus, permitting movement of blood in one direction only. Therefore, a conal valve was conceptually equivalent to a leaflet or cusp of the arterial valves of birds and mammals. The portion of the conus wall supporting the leaflet was not regarded as a valve component.

As far as is known, the study of Sans-Coma et al. [34] carried out in a shark species, the lesser spotted dogfish, *Scyliorhinus canicula* (Linnaeus, 1758), was the first to show that anatomically and functionally, each pocket-like valve is made up of the leaflet and its supporting sinus. The leaflet is the most mobile component of the valve which opens and closes during the cardiac cycle. The sinus is the hollow portion of the conus wall, whose borders support the leaflet. Since then, this notion has been adopted by several authors when describing the anatomy of the cardiac outflow tract valves of chondrichthyans [35], early actinopterygians [36], and teleosts (modern actinopterygians) [36,37,38,39,40].

In chondrichthyans (reviewed in [34]) and early actinopterygians [41], the number and arrangement of the conal valves are highly variable; both traits may even diverge between members of the same species [42]. The valves are usually distributed in several transverse rows along the conus arteriosus, which connects distally with the bulbus arteriosus (Figure 1C). The pocket-like valves are by far the most common type (Figure 1C). They play the main role of preventing blood backflow from the aorta, similar to the function of the mammalian aortic valve. An additional function of the cardiac conal valves in fish is to collaborate in the reduction of aortic pressure to protect the delicate vasculature of the gills [43]. The other, smaller valves (Figure 1C’), when present, are mere protuberances that have much less effect in avoiding flow from the conus arteriosus into the ventricle. Descriptive studies on fossilized hearts of the extinct teleost species *Rhacolepis buccalis* have shed light on the debate about the direction of evolution of the cardiac outflow tract in osteichthyans. The finding of several rows of valves in the conus arteriosus of the species *R. buccalis*, representative of a basal group of teleosts, suggests that over the course of evolution the number of valves in the conus arteriosus of teleosts has been reduced, eventually dwindling to one or two at present (Maldanis et al., 2016). Indeed, in the extant teleosts, the number of conal valves is smaller in consonance with the reduction in length of the conus arteriosus. A few species belonging to ancient groups possess two transverse rows of valves. Most teleosts, however, have a single row composed of two major pocket-like valves, which often resemble anatomically the outflow tract valves of birds and mammals (Figure 1D). One or two minor pocket-like valves are also present in several species.

## 3. The Cardiac Outflow Tract Valves of Early Sarcopterygians

In describing the cardiac outflow tract valves of the phylogenetically early groups of sarcopterygians (lobe-finned fishes), such as the crossopterygians (coelacanths), dipnoans (lungfishes), and amphibians, the concept has been usually applied that a valve is a leaflet or even any other anatomical element that might contribute to prevent the return of blood to the heart. As for the crossopterygians, exemplified by the coelacanth *Latimeria chalumnae* Smith 1939, it was briefly quoted that this species has 24 conal valves, without specifying their morphology [44].

In dipnoans (lungfishes), the concept of the cardiac outflow tract valve has been used ambiguously. The valves are located in the outflow tract portion that has myocardium in its walls. The valve shape is highly variable. There are pocket-like valves of different size, and also transverse protrusions, separated by furrows, and small incisura at the luminal side of the outflow tract, all of which have been described as valves [45,46,47,48].

The shape, size, and spatial distribution of the outflow tract valves are also notably variable in amphibians. In these animals, the valves are also placed in the myocardial portion of the outflow tract, the anatomy of which markedly differs between lung breathing and lungless species. In those having lungs, the outflow tract is partially divided into two channels, the *cavum pulmo-cutaneum* and the *cavum aorticum*, by a spiral fold that has been often termed ‘spiral valve’. The spiral fold is vestigial or absent in most lungless amphibians [49]. The other anatomical elements which have been described as valves differ in shape and size, and are usually located at the proximal and distal ends of the myocardial portion of the outflow tract [49,50,51,52]. Most often, they are pocket-like valves (Figure 1E) but other, simpler structures have also been included in the category of valves. In no case, however, has the portion of the outflow tract wall which supports the leaflet been regarded as a valve component.

## 4. The Cardiac Outflow Tract Valves of Reptiles

The sauropsids included in the classic group of reptiles have a cardiac ventricle divided partially by one or two septa, giving rise to two or three ventricular cavities, respectively. The only ventricle with a complete septum is that of crocodilians. In all cases, blood flows from the heart to the lungs through a single pulmonary trunk that divides into right and left pulmonary arteries, and to the body through two aortic or systemic arteries, right and left. The outflow tract valves are located at the base of the pulmonary trunk and at the anatomical origin of each aorta. These cardiac valves have received little attention and what can be gained from the literature is that they are usually semilunar or pocket-like in shape [53,54,55,56]. At the base of each artery there are generally two valves. Those of the pulmonary trunk have barely been studied. The valves of the aortic trunks, especially those of snakes, have been described in more detail [56]. Interestingly, these authors used the term aortic valve to refer to the set of two valves existing at the base of each aorta, thus opting for the nomenclature applied to birds and mammals.

## 5. The Cardiac Outflow Tract Valves of Birds and Mammals

In birds, namely the remaining group of sauropsids, the basal portions of the aortic and pulmonary arteries are usually composed of three valves. These two sets of valves have been termed aortic and pulmonary valves, respectively, probably because of the influence of medical viewpoints [23,57,58].

From the zoological viewpoint, mammals (Figure 1A,B), and also birds, regularly have three valves at the base of the aortic and pulmonary trunks. Each of these six valves is, in fact, an anatomical unit composed of a leaflet and its sinus. However, the resulting trivalvular aortic and pulmonary complexes, established evolutionarily in concomitance with the regression of the conus arteriosus, have been successful in performing the enormous work to which they are subject over a lifetime. In this regard, it should be noted that in humans, the presence of three leaflets of similar size constitutes the most efficient geometrical condition to prevent blood backflow to the ventricles [59,60,61]. Valve complexes built up by leaflets of dissimilar sizes or by a different number of leaflets are less efficient; they are considered cardiac anomalies or malformations by physicians, because they very often entail the risk of clinically relevant complications. For example, the presence of only two leaflets in the aortic root, which is normal in the aortic trunks of reptiles, is regarded as an anomaly from the medical perspective [62,63]. In fact, this condition, termed bicuspid or bifoliate aortic valve, is the most frequent congenital cardiac defect in humans, with an estimated prevalence of between 0.5% and 2% [64,65,66]. Considering the serious complications occurring in at least one third of cases, the bicuspid aortic valve may be responsible for more deaths and morbidity than the combined effect of all other congenital heart defects [65]. Interestingly, this example illustrates that a valvular condition that is normal and does not lead to complications in certain vertebrates, such as reptiles, may be the cause of disease in their descendants, namely in mammals.

## 6. Concluding Remarks

In extant jawed vertebrates, such as chondrichthyans, ancient actinopterygians, and early sarcopterygians, the primitive valvular system of the cardiac outflow tract is characterized by the presence of multiple valves of different shapes and sizes. The system evolved to a more simplified and regular design in those groups where the conus arteriosus became remarkably reduced in size. This is the case of the extant teleosts and amniotes. From the zoological viewpoint, the presence of three valves at the base of each great artery of the heart is the most common condition in birds and mammals.

From the medical perspective, each set of three valves constitutes a morpho-functional unit. This notion, which has been used successfully during centuries for improvements in the treatment of patients with valve diseases, and requires, in our opinion, no conceptual change. Nonetheless, it is suitable that all heart anatomists are aware of the anatomical variability of the cardiac outflow tract valves. This should help to avoid further misunderstandings between physicians and zoologists when using the term cardiac outflow tract valve.

## Data Availability

Not applicable.
